# Availability Improvements through Data Slicing in PLC Smart Grid Networks

**DOI:** 10.3390/s20247256

**Published:** 2020-12-17

**Authors:** Paul Negirla, Romina Druță, Ioan Silea

**Affiliations:** Department of Automation and Applied Informatics, Politehnica University Timisoara, 300006 Timisoara, Romania; romina.druta@student.upt.ro (R.D.); ioan.silea@upt.ro (I.S.)

**Keywords:** data slicing, Smart Grid, low-availability networks, smart metering profiling, meter firmware update, narrow-bands

## Abstract

An electrical power grid, is an interconnected network for delivering electricity from producers to consumers. Electrical grids vary in size from covering a single building through national grids (which cover whole countries) to transnational grids (which can cross continents). As the rollout of smart meters continues worldwide, there are use-cases where common solutions fail and the network availability of certain meters is very low due to poor communication conditions. This paper proposes a data slicing model for large data files which have to travel securely and reliably throughout the Smart Grid. The manuscript addresses improvements for PRIME PLC network availability by using correct data slicing at the application level along a tuned transmission rate in accordance with the noise levels of the power grid. Successful communications, even at low rates, mean that no manual interaction from energy supplier operators is needed reducing the maintenance costs for both the energy companies as well as for the end user. Experiments on a low power electrical grid setup have been performed in order to evaluate availability improvements through the proposed method as well as the feasibility of remote firmware upgrades. The results have shown that the current approach has similar upgrade time results with a manual firmware upgrade performed through an optical probe. Moreover, the results show that the presented remote firmware upgrade method is reliable and practical.

## 1. Introduction

It was considered important, from the beginning, to present the synthesis of the notations and abbreviations used. In Table 1, the notations and abbreviations are presented in the order in which they appear in the text.

The present manuscript is an extended version of previous work published in [1]. The initial concept has been extended towards analyzing methods for remote firmware upgrades in low-availability smart metering networks based on power line communication protocols.

With the rise of renewable energy technologies and with more solar and wind energy harvesting solutions being installed around the grid the need to communicate reliably with the energy power distributors is higher than ever. Smart meters enable an effective management of each household as utility companies can gather more data about the energy consumption and production, the distributors are informed automatically about any outages and new features or tariffing schemes can be provided remotely to end users. Smart meters are strongly connected along the power grid to form a Smart Grid that aims to dynamically control the imports and exports of energies in an optimized way in order to achieve a lower carbon footprint reducing costs and improving sustainability at the same time.

In order to maximize resource utilization in an efficient Smart Grid, power supply management is handled by smart metering equipment. This equipment can gather large amounts of information that need to be sent for analysis along the power lines back to an energy supplier. After the analysis of load profiles and other energy usage patterns by using novel algorithms, augmentations are sent in different formats back to the smart meters. The information at the end device can come as sets of parameters or as entire new software packages that fit the user profile at the targeted household.

In order to handle transition to a rapidly expanding Smart Grid, it is important to be able to update software remotely, such as the one seen in meters, without changing the equipment or doing upgrades manually in the field. Remote image uploading functionality enables those meters to get updated characteristics on an as-needed basis [2].

While the deployment of smart meters in Smart Grids and smart cities aim to provide more energy options and energy-saving choices for customers, the challenges of obtaining information from and to end users across the Smart Grid have been highlighted frequently in the literature as challenging task [3,4].

Smart meters can talk to their main energy providers through power lines, radio or mobile communications. It is often the case that some nodes in the smart energy grid have a low network availability, and sending a few data frames is the best that can be achieved at isolated ends of the grid or in very noisy environments.

The current paper is structured as follows: the introduction highlights the aim, the significance and the importance of the chosen topic, then the context section mentions the current state of the art and presents a quick comparison of key publications on challenges of data transmissions over power lines along the currently proposed models. The third section, materials and methods, iterates the materials and equipment used throughout the implementation of the slicing model. The methods described also cover the tests undergone for upload from smart meters towards a data concentrator and the download throughput tests needed during a firmware upgrade process. The results section covers the description of the experimental results described in the prior chapter besides their interpretation a total of 20 load profiling upload throughput tests are analyzed and 60 firmware image downloads have been performed on both the power line interface and the optical probe interface. Finally, the discussions and conclusions sections end the paper with a broad view of the implications of the current findings.

## 2. Context

The third energy package [5] requires European Union member states to ensure, for the long-term benefit of customers, the introduction of intelligent metering schemes. There are close to 60 million smart meters already installed in just four Member States [6] (Finland, Italy, UK and Sweden) and, whilst 95% [7] of the premises are reached without issues, there are going to be several thousands of households that are left in an inaccessible part of the smart energy network where standard approaches are no longer fit for purpose.

The question of how the Smart Grid security can be improved has been an active research topic in the last few years and solutions have emerged from physical layer improvements such as up to the application layer where internet connected firmware upgrades as described in [8]. The literature highlights the issues that arise from an insecure Smart Grid and the risk it poses by being allowing attackers to destabilize a grid by having remote control to key nodes. This paper aims to improve the security of low-availability nodes by allowing every node to get remote updates in a practical and timely manner. Attackers can decrease the network bandwidth or obtain the traffic including private data such as search histories, login information, and device usage patterns by exploiting the vulnerabilities in firmware upgrades to install malicious firmware [9]. Mlynek et al. [10] show the data rates of different power line communication protocols used in smart metering, but it concludes that all PLC will suffer from nodes being not close enough to their peers (i.e., the attenuation of the power line is proportional to the length of the power line) as well as from the power quality of the grid. The proposed solution of having repeaters at certain distances is not feasible in our case, as we aim to not add any new equipment on the grid and to only use what has already been deployed in the field.

In [11], Andreadou underlines the decisions made by major European power distribution firms during their Smart Grid expansions, with PRIME being one of the most common choices due to its reduced installation costs in the low voltage grid. Moreover, it is expected that around 200 million smart meters will be installed by the year 2020. There are more than 50 European projects that are directly or indirectly linked to smart metering applications all of which need to be maintained over a prolonged period of time, which implies firmware upgrades and network availability. Moreover, the parallel rollouts without a synchronized protocol has called attention to a more standardized approach. In [12] IEC 61850 communication standard is recommended for communication in Local Area Network (LAN) and eXtensible Messaging and Presence Protocol (XMPP) in Wide Area Network (WAN). This partially solves the common standardization issue but still suffers from latencies up to several minutes as well as from very low bandwidths.

A literature review of the Smart Grid cyber security performed by Baumeister [13] highlights the risk of having smart meters that will no longer benefit from security upgrades on the network due to availability issues. Smart meter infrastructure surveys [14,15,16,17] call attention to the security risk posed by reduced availability and offers solutions ranging from VSAT [18] that support high range coverage or LTE and mobile solutions [19] with availabilities close to 99.9%. The issue is that the meters are already installed and adding new technologies would not be feasible cost wise.

We have to work at the application layer with the little availability we see on those already installed meters to ensure that every bit of communication counts and retransmissions are undesirable.

For the electricity market in Romania, the chosen deployment model has a middleware layer with data concentrators connected by wire to the smart meters. Communication of data is made through Power Lines Communication (PLC) wiring from the meters to the concentrators and through various communication ways from concentrators to the central application. The national rollout plan aimed for 80 percent coverage in the country by 2020 and complete coverage by 2022. As for the chosen communication protocols so far, most of the market uses PLC, M-Bus, WiFi, RS-485, cellular network. PLC systems have a divided market between the G3 and PRIME Narrow-Band PLC protocols [20]. The latter technologies, while they have great advantages of not requiring new wiring during deployment, suffer from interference generated by consumer equipment or from large distances between households. The chosen equipment requirements by ERBD and the Romanian Authority for Energy Regulation are robust and reliable in high density areas but the limitations on wiring network and low throughputs of the chosen protocols become challenging when we need to deal with large amount of data that needs to be moved across the grid. While the deployment of the smart meters has not finished yet, the requirements are already at the upper limit of the grid.

The DLMS/COSEM (IEC 62056, EN13757-1) global standard for smart energy metering already requests certain data structures such as tariffs that can change dynamically every other minute or support for multiple energy suppliers and most importantly load profiling of all supported tariffs for all supported contract at a resolution of 1 min for all types of energies (active, reactive, imported/exported and by quadrant). Such complex structures usually overwhelm the chosen protocols and can easily reach sizes of several megabytes. On the other hand, performance evaluations of the two narrowband PLC systems (PRIME and G3) show that this transmission can be achieved at a greater speed on PRIME but it behaves worse with noise interference [21,22].

Packet error rate in PRIME networks increases with packet size as proved by Farias in [23]. PRIME meters registering almost zero availability as packet sizes increased significantly. Hardware solutions have been provided and observations made in the literature are valuable, but this does not improve matters for already deployed meter that encounter significant packet loss. Therefore, the current manuscript aims to improve packet error rates which subsequently could lead to more successful remote firmware upgrade deployments.

This can be improved by using the correct data slicing and hybrid communication distribution across multiple protocols as proposed below.

We proposed a method for increasing the availability of smart meters connected through power line communication PRIME protocol. The smart meters that rarely keep a solid network connection with the energy distributor were able to be remotely updated providing security updates in a practical time window. Using this approach, distributors can avoid having manual operators on field running readout operations or manual smart meter firmware upgrades. This can result in lower maintenance costs from which both the energy providers and the end users can benefit.

## 3. Materials and Methods

To reach the goal of having successful PLC communications with low availability smart meter nodes, our solution needs to avoid retransmissions, to have a high tolerance on erroneous transmitted packets and to be capable of assembling back the initial file whenever we detect a complete transmission. Slices might be delivered on separate communication channels where those are available on the smart meter itself or on neighboring peers.

For this application we have used the chosen meter technologies required by the regulation authorities: PLC communication using PRIME protocols applied on ST smart-metering evaluation hardware kit EVL-KSTCOMET10-1 based on a Cortex-M4 core. The default configuration is fit for PRIME (ITU G.9904) CENELEC A-band protocol standards and the PLC line coupling interface is designed to allow the device to transmit and receive on the AC mains line using any narrow-band PLC modulation (single carrier or OFDM) up to 500 kHz [24]. The end points are connected in series and the network is controlled by a third board acting as a PRIME concentrator. All devices are fit with a W25Q16JV external flash for storage of 16Mbits of load profiles in DLMS formats.

Each type of network has a maximal amount of data that can be moved across nodes at any given time. In computer networking, the maximum transmission unit (MTU) is the size of the largest protocol data unit (PDU) that can be communicated in a single network layer transaction. Depending on the chosen method of transport, the data slicing approach needs to account for the transport type constraints. In a typical Ethernet v2 connection, an MTU of 1500 bytes is expected [25] and whenever PRIME and other interfaces are merged together (most commonly with ethernet at the concentrator level) the MTU considerations need to be made by having a complete overview of the protocols and physical environments involved in the data communication from one end of the grid to the other.

Although larger packets mean fewer transmissions and less traffic used for overhead transportation, there are certain tradeoffs that come with bigger chunks of data being transmitted at once. If we are using a link with a slow speed of processing a large packet, will keep the network busy for a longer time, reducing the availability of those nodes till transmission is complete. Another known issue of larger packets is that even slight errors detected in large packets will cause the entire frame to be retransmitted. In environments that are prone to data corruption, smaller data slices will be used instead.

**Figure 1:**
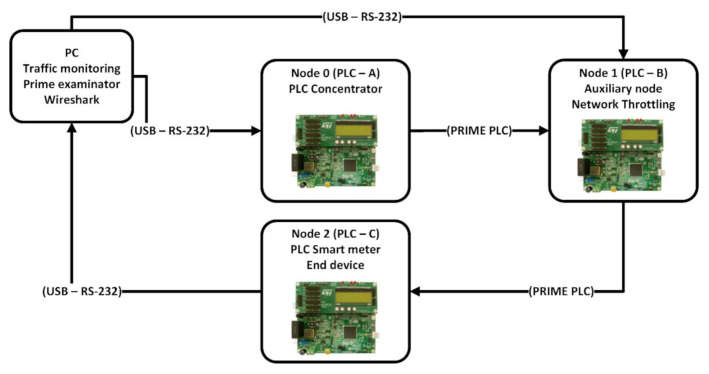
PoweRline Intelligent Metering Evolution (PRIME) data slicing test network topology.

**Figure 2:**
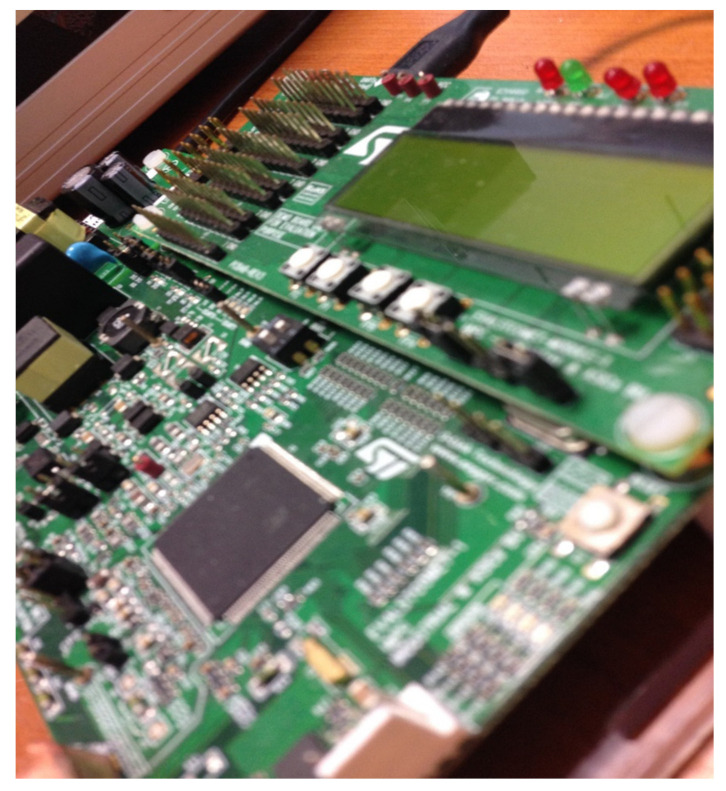
STMicroelectronics (ST) power-line communication (PLC) Evaluation smart meter board installed close-up with USB built-in debugging capabilities.

The proposed solution consists of slicing large data packets into chunks that are equal to the smallest MTU in the communication chain allowing one packet to reach the destination in a reliable manner, before a second transmission is initialized. This way we ensure there is no bandwidth taken by the retransmissions of an entire large structure that has been segmented by the low layer protocols throughout the power lines transmission. Figure 1 pictures the network topology for the slicing solution examination. This topology consists of three separate power line nodes based on the PRIME evaluation kit from ST electronics, EVL-KSTCOMET1-1 [26], connected to the same electrical socket whilst all nodes are also connected to a monitoring PC through their built-in USB interface. The USB interface uses the custom application written for this layout to control the network quality or to examine the transported frames. Node 2, PLC-C will be examined as the device under test whereas the other nodes have an auxiliary role. Node 0 runs a generic PLC PRIME concentrator application maintaining the mesh node and Node 1 uses its application to recreate a node that drops packets at a given rate to mimic real world conditions for poor performance networks [27]. In Figure 2, a close up of the device under test can be seen with the USB connection that provides both JTAG debugging interfacing as well as Universal Asynchronous Receiver-Transmitter serial access on a common bridged interface.

**Figure 3:**
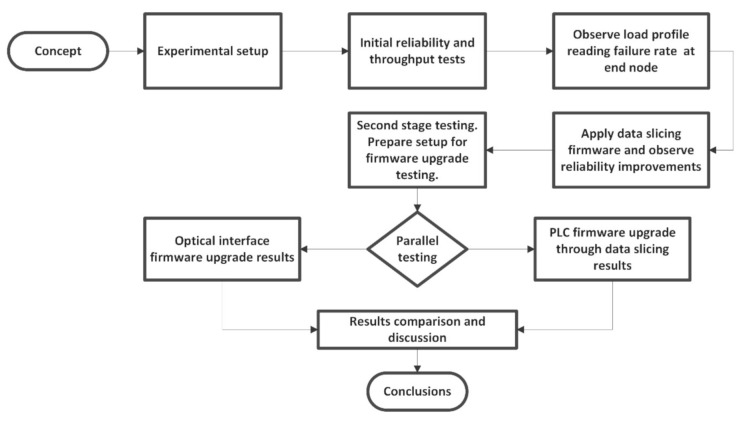
Flow chart diagram of the proposed method and its testing methods.

A flow chart diagram of the chosen testing approach is represented in Figure 3. It contains the steps undergone throughout the experiments and a quick overview of how the results have been analyzed.

### 3.1. Packet Structure

Each block of data being sent over the network is preceded by an overhead called a data slice header. A packet has two sections for each layer: the header and the body. The header provides protocol details specific to the network, while the body comprises data for the node, mostly made up of an entire packet from the next network. The details derived from the layer above each layer are viewed as data and its own heading refers to the above data. This cycle of data protection is regarded as encapsulation when adding a new header.

In our protocol, the body contains the updated binary information in one form or another and the header provides sufficient information for the receiver to be able to process, validate and control the reconstruction of the end file. This is commonly achieved by appending a sequence ID and a total number of slices to each individual data slice.

PRIME v1.3.6 used in currently deployed smart meters uses differential modulation with one of three possible constellations: DBPSK, DQPSK or D8PSK. If the channel environment is good and no ½ rate convolutional coding is required, it can support speeds up to 47 Kbps, 94 Kbps and 141 Kbps respectively. Table 2 pictures the raw data speed expected in a common PRIME network [28].

Given that those throughputs are ideal, and the amount of overhead is significant, moving the large data blocks around the network will cause fragmentation throughout the entire OSI layers. The current smart-grid implementation looks as follows in Table 3:

D8PSK modulation with no convolutional code has been chosen to achieve the highest PRIME throughput with an MSDU = 2268 bytes. To avoid having any segmentation and reassembly on PRIME layer, we need to look at its Common Part Convergence Sublayer, CPCS. The CPCS functionality is responsible for splitting outgoing data into ClMTU constant segments of 256 bytes each. PRIME 1.3.6 supports up to 64 segments of ClMTUSize. This leads to a maximum transmission length of 16,384 bytes after which upper layers need to handle further segmentation and reassembly.

Segmentation at application level will follow the same overhead structure as the PRIME Phy Common Part Convergence Sublayer as described in the R1.3.6 standard in Table 4:

In order to define a fit N number of supported segments, we will look at one of the most common failed transmissions in poor smart PLC networks, which consists of retrieving the energy load profile of the previous day with a resolution of 5 min. Each load profile entry consists of total energy register values that cover both imported and exported active electrical energy as well as all reactive energies on all four quadrants. The numbers of supported contracts and supported tariffs number have been chosen as 6 different contracts on 16 different tariff schemes to mimic the features supported by the current DLMS certified meters.

$$\begin{array}{l} LoadProfileDaySize\\ \;=NumTypesOfEnergyRegisters*RegisterSize\\ \;*NumOfSupportedTariffs*NumOfSupportedContracts\\ \;*MinTimeResolution \end{array}$$

LoadProfileDaySize=6∗8 bytes∗16∗6∗288

LoadProfileDaySize=1,327,104 bytes

$$NSegs_{min}=LoadProfileDaySize/{\mathrm{ClMTUSize}}_{*}$$

$$NSegs_{min}=1327104\;bytes/256\;\mathrm{bytes}$$

$$NSegs_{min}=5184$$

Considering the above a number N = 16 bits will suffice, leading to an overhead of 34 bits for segmentation logic.

To achieve the highest throughput, all tests are run using D8PSK modulation and coding with no convolutional code.

### 3.2. Desfining the Load Profile Upload Experminets through Data Sliced PRIME Nodes

We have run the proposed solution in a simulated residential building, using an evaluation data concentrator connected through power lines to DLMS PLC PRIME boards pictured in Figure 2 by using the Figure 1 topology. In order to simulate the power line communication conditions from a low power electrical distribution grid which encounters power quality disturbances, an Integra GP 3050/3 phantom load supply has been used to power the devices under test at voltages varying in the [180 V, 230 V] AC range. As the chosen development kit is not battery operated, its VIPER26H power supply can provide the required voltages for the communication controller even during power sags. Power dips are usually present in an unbalanced grid at a remote location, and a theoretical power dip for a prolonged period of time at a voltage level of 180V has been chosen as a worst-case scenario in the experimental low voltage distribution grid, whereas 230V is the nominal voltage. The experimental low power electrical distribution grid setup had no intentional harmonic distortions applied and there was no other equipment powered from the low voltage lines aside from the devices under test. In this environment, the chosen modulations and coding could reach a theoretical speed of 128.6 kbps but the size of the daily load profile and the noise on the network made the data transfer impractical.

By using data slicing with our proposed pattern, data transfers became reliable with complete load profiles being transferred from PLC-C PRIME meter board back and forth to PLC-A PRIME concentrator through PLC-B PRIME meter. By choosing slices close to MTUs of Figure 2 layers, the following tests have been carried out:

Test 1. Initial test throughput with an emphasis on the rate at which slices needed retransmission during a normal transmission of a Daily Load profile reading that has a size LoadProfileDaySize = 1,327,104 bytes.

Test 2. Sliced test with the transmission of a Daily Load profile reading configured for a data slice size = 256 bytes. This will split the formatted data into 5184 slices.

### 3.3. Firmware upgrade with data sliced PRIME communication feasibility experiments

Given the preliminary results obtained by the data sliced load profile download, we needed to consider the impact of a similar approach in a remote firmware download operation as well as in a post-production firmware upgrade where a PLC upgrade is more convenient than a manual update through the optical interface. For these tests, a generic ST-COM-based meter has been used to compare the throughputs of data-sliced PRIME firmware upgrade download process operations against a conventional IEC 62056-1-0:2014 download through a high-speed galvanically isolated infrared optical communication.

This is a single node test and does not cover strategies for multi-node [29] smart metering networks nor the drawbacks of adding security measures [30] in the overall upgrade process but, both of these will be considered for further research.

As the devices under test use a 1 MB internal flash, we will have to simulate a complete upgrade that fills the entire memory as this covers the scenario of a partial upgrade as well for meters that support firmware separation features. All tests have been run under the same conditions described in the previous load-profile upload iterations and from the smart meter upgrade steps only the image transfer steps (i.e., Step 1—Get Image Block Size, Step 2—Initiate Image transfer, Step 3—Transfer Image Blocks) from [31]) have been considered for a complete test result.

**Figure 4:**
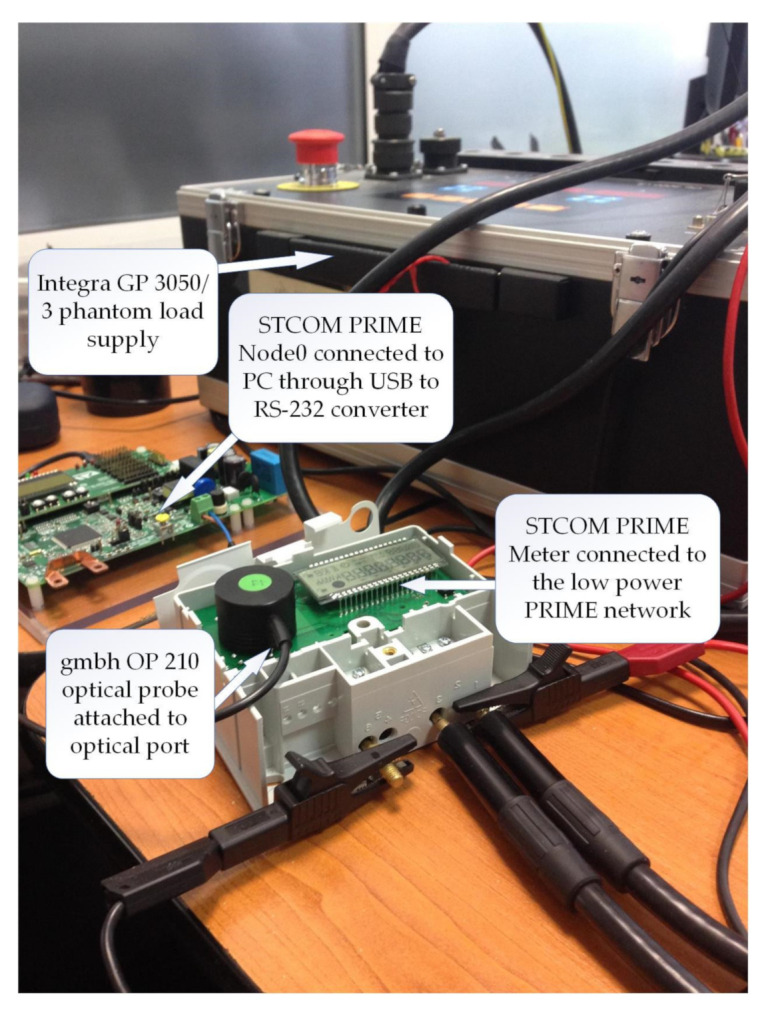
STCOM smart meter connected to PLC concentrator and to optical probe concomitantly.

Figure 4 shows the test environment with the PRIME meter connected to both a STCOM PRIME node as well as to an optical probe. Optical transmissions have been performed through the standard COSEM IEC 62056-1-0:2014 protocol by using a gmbh—OP 210 USB probe at its maximum speed of 19200 bits/s. The application layer is identical and has not been changed between testing the optical interface and the electrical one, respectively.

## 4. Results

By running COSEM commands through the setup described in the previous section, we have gathered results for 10 unique load profile readings from the same node without using data slicing and with slices segmented at 256 bytes. Each time the protocols have lost a packet in the PRIME transmission, a retry is marked in the monitoring tool and is then marked as a packet that was not transmitted on the first attempt. This is very important as packet loss is the main metric the protocol aims to improve, and throughput is not essential when nodes are barely reachable.

**Figure 5:**
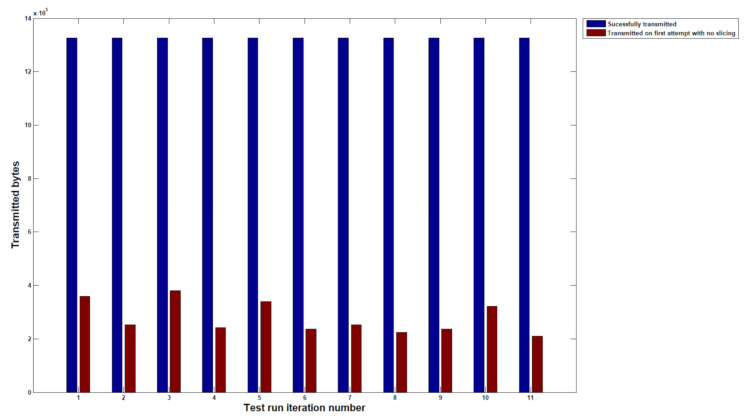
Initial results on daily load profile reading over PLC-PRIME from PLC-A to PLC-C.

**Figure 6:**
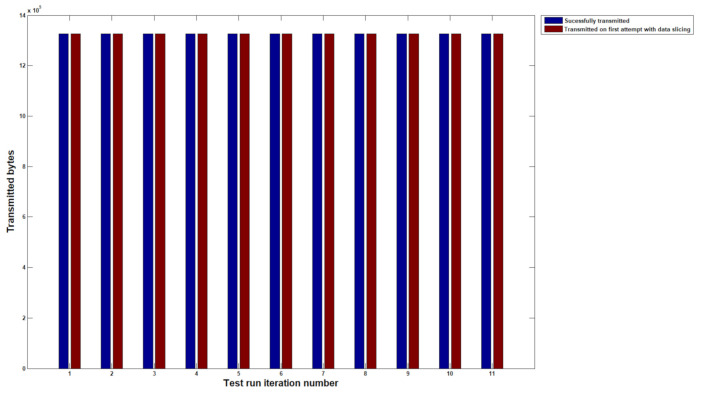
Transmission of daily load profile readings with confirmation on receiving with no retransmissions required.

Before proceeding to any solution testing, we need to measure how the black box network throttling on Node 1 behaves. Figure 5 shows that a mean retransmission rate of 13% is required by analyzing the PLC Wireshark traces. This result is fit for purpose and the Figure 1 topology can be used further to analyze how a low availability Node 2 device operates with a timed communication with CIMTUSize sized slices. In Figure 6, the data showed no retransmissions being required throughout the communication with Node 2 during the load profile read command on ten separate iterations. The outgoing load profiles have successfully reached the targeted destination with no packet loss. Moreover, each slice has been properly transmitted in a single transmission window with no retries. The throughput, however, diminished significantly during our experimental analysis; therefore, this has been looked at in the next steps.

**Figure 7:**
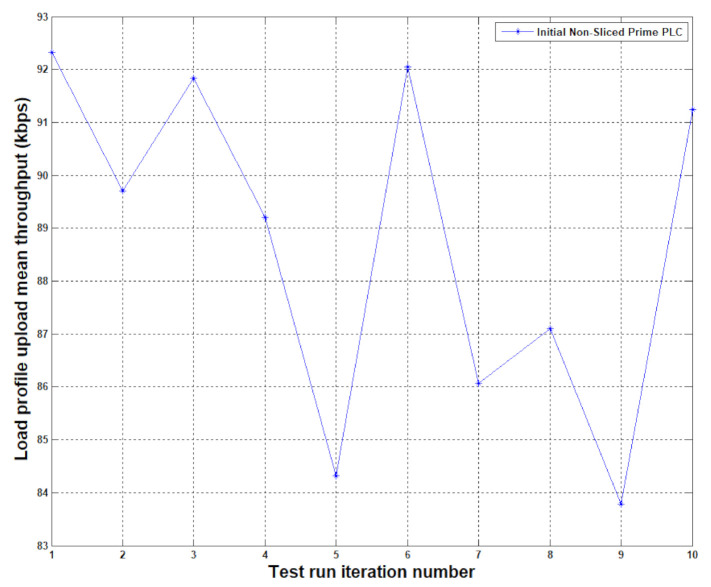
Thresholds observed on unreliable PLC communication channel with no data slicing at application level.

Figure 7 shows our throughput results with the non-sliced PRIME communication, with a direct connection to Node 2, which achieved throughputs in the [83 kbps, 93 kbps] range which is not unusual for a healthy power line network. Aiming for a network with no retransmissions, as a clean communication would look like, we analyzed the impact of the paced solution with data slices hitting the end device in one go, but at a visibly slower rate. For devices that would stay for a longer period of time at a 0% availability level, throughput is not an immediate issue, as these devices would be unreachable for a long period of times anyway so having the communication fragmented over shorter periods of availability rather than a long window fractured by down times and packet loss.

**Figure 8:**
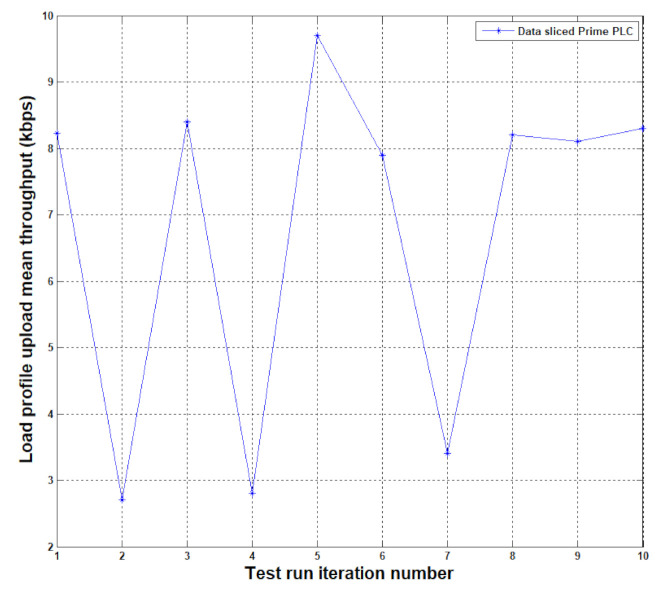
Thresholds observed on PLC-A to PLC-C communication channel with data slicing at the application level.

Finally, Figure 8 shows the cost of having a more paced transfer of slices, the throughput went from a mean value of 88.761 kbps down to 6.763 kbps, allowing a full day profiling to be transmitted in about 20 min in the most exhaustive format.

The throughput achieved by keeping the segments of communication handled by the so-called data sliced application is enough to ensure the smart device will achieve enough successful transmissions so that the energy provider has enough profiling data to support an optimized tariff scheme. The billing can be automated even for those devices as the load profile is together with the images for software updates the largest chunks of information that need to travel throughout the grid. The application needs to be able to adapt however to this mode whenever it is necessary, and it can be a fail-safe mechanism that the nodes might rely on as a last resort.

Moving forward we need to analyze if this sliced method is fit for firmware upgrades as the security surveys by Baumeister [13] and Asghar [14] point out to be the most critical step in a smart meter environment. As devices with no availability are commonly manually updated by an operator in the field at the location at which the smart meter is installed, we have compared the upgrade process in the network with a manual upgrade through the optical probe interface. If this achieves successful results, it means that weak links that were left unsecured in the meshed network will be able to receive new firmware containing security patches as well as new features and optimized software tailored for a particular unit.

Table 5 shows the experimental iteration of the 1MB firmware image file download through the PRIME, data sliced, power line communication as well as the throughputs obtained by the optical interface on the same file. During the test iterations pictured by Table 5, the optical firmware upgrade throughput averaged a speed of 15016.3 bps (14.66 kbps) whilst the data sliced PLC approach obtained an average speed of 7769.4 bps (7.59 kbps).

**Figure 9:**
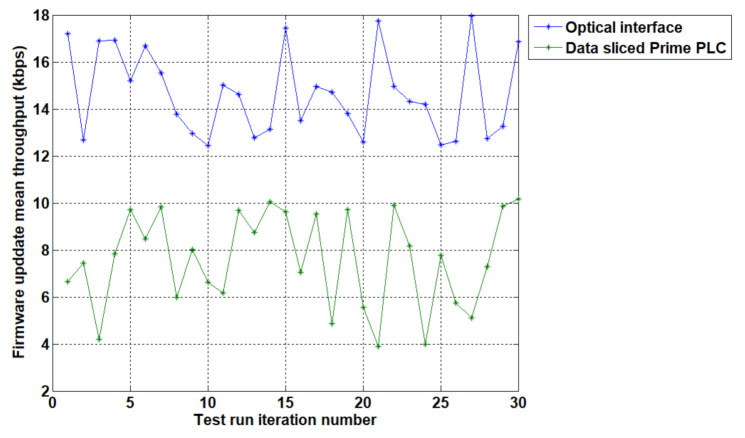
Visual representation of the firmware upgrade throughput test results for 1 MB files through both optical and sliced PRIME PLC communication.

A visual comparison of the throughputs is depicted in Figure 9, whilst it shows the optical the interface running at slightly better throughputs, we have shown that the data sliced approach is still a viable solution with throughputs in a similar value range.

## 5. Discussion

As the results from the prior chapter demonstrate, by reducing the transmitted segments in a given time window, we have shown that the retransmission rate has been significantly reduced and the data sliced PLC communication can improve availability by getting at least one segment on the targeted device whenever a brief availability window exists. Moreover, the firmware upgrade process can still achieve throughputs that are comparable to a manual operation performed by a field engineer without the costs of logistics that this method involves. This means that nodes that are at the end of the meshed grid in a PRIME network can be updated and are no longer left unsecured due to the unavailability of getting an update finished in the same availability window.

Table 6 shows a statistical analysis of the experimental data and it outlines the mean throughput of 7.59 kbps for data sliced PRIME devices with a sample standard deviation of 2.02 and a confidence interval (95%) between 6.86 kbps and 8.31 kbps.

The tested throughput of the downloaded files is comparable to a manual upgrade performed by a field engineer through a galvanically isolated optical probe making a PLC upgrade approach closer with the current industry needs.

The comparison of this approach against dedicated solutions surveyed by literature in [32] emphasis that any technology that improves the overall availability makes the Smart Grid better and more secure. Any system cannot be more secure than its weakest link therefore adding more approaches to the smart metering world toolbox can guarantee a complete solution in the end.

Network topologies of different types, including more flexible radio technologies, are competing for bandwidth in a cost-effective manner although interferences are expected; therefore, we need to keep an eye on both throughput and long-term system availability [33].

There are few standards at the moment that cover more than one main of communication across the Smart Grid. Rolling out a data sliced pattern or similar one can currently only be approached by specific manufacturers and having different brands of meters on the smart network cannot work in a standardized way. The standards need to become more flexible allowing new technologies to be implemented at lower OSI layers rather than having manufacturers to rely on their application-level implementation. In the end, a Smart Grid that can benefit from all the advantages of different types of data links can create a hybrid solution where a meshed network can benefit from smaller improvements such as the one presented above to create more comprehensive multi node/multi layered communications such as the ones presented in [29,33,34,35].

## 6. Conclusions

In this paper, we proposed a method for increasing the availability of smart meters connected through power line communication PRIME protocol. By increasing the availability, the power supplier can reach a larger number of smart meters and energy consumption profiles such as daily load profiles can be gathered reliably. More importantly, the smart meters that rarely keep a solid network connection with the energy distributor can be remotely updated providing security updates in a timely manner. This means that manual field operations for upgrading smart metering equipment or for manual meter readouts in remote locations with poor network connectivity can be averted resulting in lower maintenance costs, from which both the energy providers and the end users can benefit.

Results from experimental trials on smart meters running the above-described model for data slicing in Smart Grid environments with low-availability have proven the approach to be working correctly. By using data slicing with our proposed pattern, data transfers became more stable with no need for retransmission attempts all at a cost of throughput. In addition, devices scattered around an unreliable network have seen a noteworthy increase in successful transmission of large profiling data frames as well as in successful firmware upgrade deployments.

Based on the need for a more secure low power electrical distribution grid, the concept of improved firmware upgrades methodologies for smart metering equipment is a key topic that needs to be followed up in future research work. It will be important that the current data slicing transmission unit size is investigated and perhaps optimized based on the quality of the communication channel. Another desirable approach would be the improvement of availability in smart meters that benefit from multiple physical transmission media of communication and how these can be joint to achieve better smart metering networks through a network fusion approach.

At a larger scale, the chosen technologies for a national rollout should be well analyzed before imposing a single solution to entire industry and the actors need to have a common strategy to overcome physical challenges encountered in the real world. The roll outs in the analyzed countries all achieved availabilities that show real improvements for three quarters of smart meter end users, but this needs to be improved by looking at software improvements as well as new technologies that can mesh with the existing ones providing a good cost per unit and an improved user experience for every household. In the end, the electrical companies and smart meter manufacturers cannot solve all issues on their own and further research towards a joint standardization is necessary. By merging current approaches with other improvements, such as the one demonstrated, can create through remote software upgrade and automatic meter readings a more secure smart metering grid with high availability devices and low maintenance costs implicitly allowing the user to have a custom firmware in any household that is fit for his energy consumption behavior.

## Figures and Tables

**Table 1:** Notations and abbreviations.

No.	Abbreviation	Meaning
1	PRIME	PoweRline Intelligent Metering Evolution
2	PLC	Power-line communication
3	UK	United Kingdom of Great Britain and Northern Ireland
4	VSAT	Very-small-aperture terminal
5	LTE	Long-Term Evolution
6	M-Bus	Meter-Bus
7	Wi-Fi	Wireless network protocol, based on the IEEE 802.11 standard
8	ERBD	European Bank for Reconstruction and Development
9	DLMS	Device Language Message Specification
10	PHY	Physical communication layer
11	COSEM	Companion Specification for Energy Metering
12	RS-232	Recommended Standard 232–Interface EIA (From Electronic Industries Alliance Standards)
13	ITU	International Telecommunication Union
14	G3	Orthogonal Frequency Division Multiplexing (OFDM) based PLC technology
16	ST	STMicroelectronics
17	CENELEC	French: Comité Européen de Normalisation Électrotechnique; English: European Committee for Electrotechnical Standardization
18	AC	Alternating current
19	OFDM	Orthogonal Frequency Division Multiplexing
20	PDU	Protocol data unit
21	MTU	Maximum transmission unit
22	USB	Universal Serial Bus
23	JTAG	Joint Test Action Group
24	IEC	International Electrotechnical Commission
25	MSDU	Media Access Control Service Data Unit
26	CPCS	Construction Plant Competence Scheme
27	OSI	Open Systems Interconnection

**Table 2:** PHY data rate and packet size parameters, for various modulation and coding schemes.

Modulation and Coding	DBPSK	DBPSK	DQPSK	DQPSK	D8PSK	D8PSK
Convolutional Code (1/2)	On	Off	On	Off	On	Off
Raw data rate (Kbps approx.)	21.4	42.9	42.9	85.7	64.3	128.6
Maximum MSDU length with 63 symbols (in bits)	3016	6048	6040	12,096	9064	18,144
Maximum MSDU length with 63 symbols (in bytes)”	377	756	755	1512	1133	2268

**Table 3:** PRIME smart energy meters Open Systems Interconnection (OSI) layers correspondents.

OSI Layer	Smart Metering PLC Layer
Application	Data slicing implementation through DLMS
Presentation	COSEM
Transport	TCP/UDP
Network	IPv6
Data Link and Phy	PLC PRIME

**Table 4:** Segmentation header fields and structure.

Name	Length	Description
Type	2 bits	Type of segment. 0b00: first segment; 0b01: intermediate segment; 0b10: last segment; 0b11: reserved
NSegs	N bits	‘Number of Segments’—1.
SEQ	N bits	Sequence number of segment.

**Table 5:** Firmware upgrade throughput test results for 1MB files through both optical and sliced PRIME PLC communication.

Test #	Optical (bps)	Optical (kbps)	Sliced PLC (bps)	Sliced PLC (kbps)
1	17,622	17.21	6813	6.65
2	12,978	12.67	7624	7.45
3	17,300	16.89	4301	4.20
4	17,313	16.91	8016	7.83
5	15,566	15.20	9933	9.70
6	17,091	16.69	8670	8.47
7	15,893	15.52	10,079	9.84
8	14,091	13.76	6142	6.00
9	13,276	12.96	8201	8.01
10	12,738	12.44	6787	6.63
11	15,369	15.01	6311	6.16
12	14,977	14.63	9926	9.69
13	13,064	12.76	8959	8.75
14	13,458	13.14	10,287	10.05
15	17,860	17.44	9851	9.62
16	13,827	13.50	7223	7.05
17	15,302	14.94	9774	9.54
18	15,062	14.71	4968	4.85
19	14,129	13.80	9932	9.70
20	12,904	12.60	5680	5.55
21	18,152	17.73	3987	3.89
22	15,318	14.96	10,131	9.89
23	14,678	14.33	8379	8.18
24	14,550	14.21	4076	3.98
25	12,783	12.48	7966	7.78
26	12,912	12.61	5866	5.73
27	18,391	17.96	5222	5.10
28	13,046	12.74	7469	7.29
29	13,565	13.25	10,098	9.86
30	17,274	16.87	10,411	10.17

**Table 6:** Statistical analysis of throughput test measurements.

Measurement Interface	Mean (kbps)	Sample Std. Deviation	Sample Variance	Coefficient of Variation	Confidence Interval (95%)
Optical	14.66	1.8064	3.2632	0.1231	14.01 to 15.31
Data sliced PRIME PLC	7.59	2.0214	4.0864	0.2664	6.86 to 8.31
